# Phase‐Changing Vanadium Oxides for Electromagnetic Radiation Management

**DOI:** 10.1002/smsc.202500614

**Published:** 2026-01-29

**Authors:** Mohammad Taha, Torben Daeneke, Sumeet Walia

**Affiliations:** ^1^ Department of Electrical and Electronics Engineering The University of Melbourne Victoria Australia; ^2^ School of Engineering RMIT University Victoria Australia

**Keywords:** adaptive, electromagnetic modulation, phase‐change, vanadium oxide

## Abstract

Vanadium oxides and their polymorphs are transforming electromagnetic radiation security in communications and infrastructure. This arises from their broadband response and potential for wavelength attenuation across the ultraviolet, optical, infrared, and radio regions of the electromagnetic spectrum. More specifically, monoclinic vanadium dioxide's sharp, reversible insulator‐to‐metal transition near room temperature enables ultrafast, tuneable switching of conductivity and optical properties, triggered by thermal, optical, or electrical controls. Chalcogenide phase‐change materials require high crystallisation temperatures and nanosecond switching times, whereas VO_2_'s volatile Mott transition operates near ambient conditions with femtosecond response and cycling stability exceeding 100 million cycles. This dynamic modulation supports real‐time absorption, shielding, and beam steering across terahertz, infrared, and radiofrequency domains, with demonstrated absorption rates tuneable from 2% to 100% and bandwidths up to 6.35 THz. VO_2_ metasurfaces offer polarisation insensitivity and multifunctionality, protecting against jamming, interception, and signal leakage. Advances in large‐area synthesis, nanostructuring, and durability have enabled both highly sensitive sensors and long‐lived smart coatings. These findings position vanadium oxides as transformative materials for physical‐layer electromagnetic security in wireless communications, infrastructure protection, and smart sensing systems.

## Graphical abstract

Vanadium dioxide's reversible insulator‐to‐metal transition near room temperature enables ultrafast, broadband electromagnetic modulation from ultraviolet to radiofrequencies. With femtosecond switching speeds and cycling endurance exceeding 100 million cycles, VO_2_ metasurfaces achieve tuneable absorption, beam steering, and polarisation control. These volatile phase‐change properties position vanadium oxides as transformative platforms for physical‐layer electromagnetic security, adaptive shielding, and reconfigurable photonic systems.

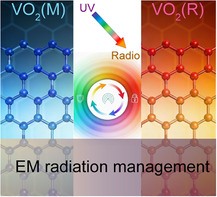

## 1 Introduction

Securing electromagnetic communications and infrastructure is a critical priority as wireless, optical, and sensing technologies evolve in complexity and bandwidth. The modern landscape integrates essential services, advanced communications platforms, and emerging fields such as quantum and smart city applications, all relying on robust electromagnetic control. Traditional shielding materials and static absorbers are limited by narrow frequency ranges and an inability to respond to real‐time threats such as jamming, interception, and signal leakage. These vulnerabilities expose vital systems to dynamic attack and manipulation, creating an urgent need for agile, tuneable, and adaptable solutions across wireless communication, physical security, imaging, and smart infrastructure [1, 2, 3, 4, 5].

The imperative for effective electromagnetic security extends beyond conventional cryptographic and software‐level defences. Today's threat landscape requires hardware‐level, reconfigurable protection systems able to counter evolving challenges such as advanced jamming, deliberate eavesdropping, and malicious interception. In this context, vanadium dioxide (VO_2_) has gained recognition as a multifunctional platform due to its sharp, reversible insulator‐to‐metal transition (IMT) near room temperature. This transition arises from strongly correlated electron‐lattice interactions and can be triggered by thermal, optical, electrical, or mechanical stimuli. The transition enables programmable modulation of optical and electrical properties over several orders of magnitude, giving materials and devices real‐time control over electromagnetic environments at the physical layer [6, 7, 8].

Vanadium dioxide distinguishes itself from other phase‐change materials through advantages that converge near room temperature. Its transition occurs at a tuneable ∼68°C (340 K), achieves IMT switching in 26–75 fs, and supports cycling endurance exceeding 10^8^–10^9^ events, combining ultrafast response with practical operating conditions [9]. Recent work demonstrates absorption bandwidths up to 6.35 THz with tunability from 2% to 100% across terahertz to infrared domains, enabling real‐time absorption, shielding, and beam steering in metasurface architectures. In contrast to non‐volatile chalcogenide phase‐change materials, VO_2_'s volatile Mott transition passively recovers without energy‐intensive reset pulses, making it inherently suited to adaptive electromagnetic systems that require continuous, reconfigurable modulation [10, 11, 12, 13, 14].

With the rise of programmable cybersecurity systems, there is a pressing need for codevelopment of advanced physical materials and smart surfaces. Only by synchronising digital and material‐level solutions can we address a diverse and rapidly shifting landscape of security threats. Vanadium oxides and their phase‐change mechanisms are positioned to fill this gap, providing robust, dynamic, and multifunctional architectures for adaptive shielding, secure signal processing, and stealth performance in variable operational settings [15, 16, 17, 18, 19].

In contrast with previous reviews that have largely focused on thermochromic smart windows, generic VO_2_ modulation, or phase‐change photonics, this article places particular emphasis on VO_2_ and related vanadium oxides as a platform for adaptive electromagnetic security. The discussion spans ultraviolet to radiofrequency operation, linking microscopic phase‐transition physics to synthesis, metasurface design, and system architectures for jamming suppression, channel randomisation, and tamper‐resistant hardware. By explicitly contrasting volatile VO_2_ with non‐volatile chalcogenide phase‐change materials in the context of physical‐layer security, the review aims to provide a unified framework for selecting and engineering phase‐change platforms in emerging secure communication and infrastructure applications.

## 2 IMT in Vanadium Oxides

### 2.1 Ultrafast Dynamics and Physical Changes

**FIGURE 1:**
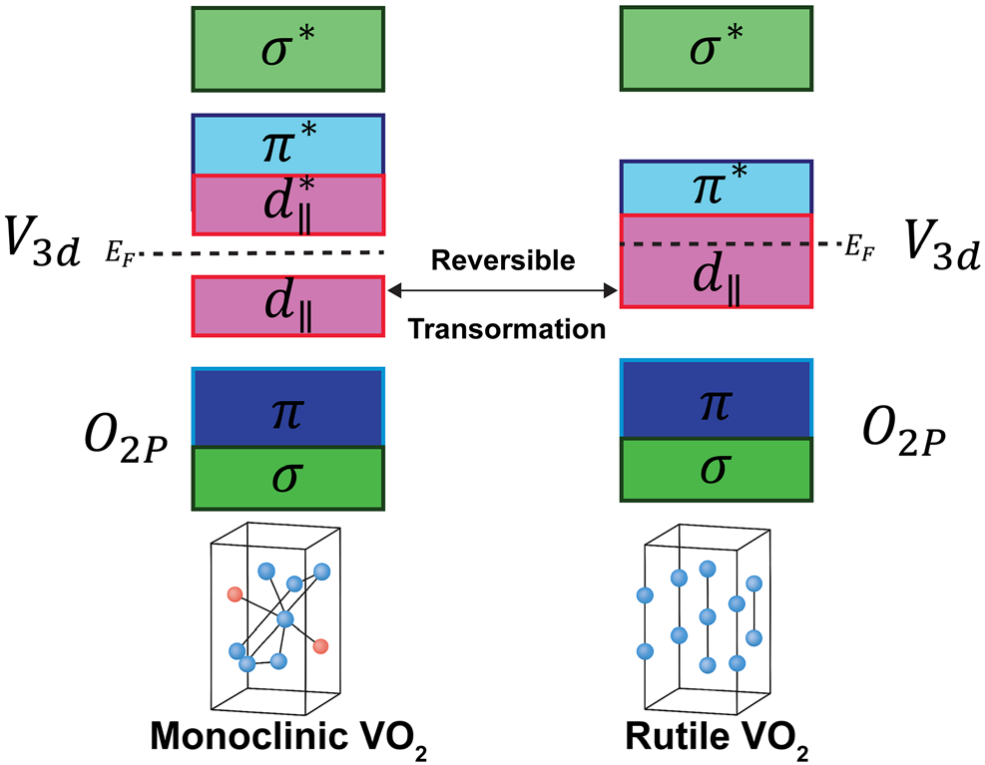
The dramatic structural transformation of VO_2_ and the bandgap collapse between the V 3*d‖* and 3π* states.

The IMT in vanadium dioxide (VO_2_) is a canonical example of a strongly correlated phase transition, involving simultaneous changes in lattice structure and electronic band behaviour on ultrafast timescales. Under femtosecond laser excitation, reports show that the transition can occur in 10–100 femtoseconds, coupling the rearrangement of atomic positions with a collapse of the electronic bandgap [20, 21]. This transition in VO_2_ is driven by a dramatic structural transformation as seen in Figure 1, the material shifts from the monoclinic to the rutile phase, causing the bandgap between the V 3*d‖* and 3π* states (approximately 0.7 eV) to collapse. This transformation leads to pronounced electronic and optical consequences.

In its insulating monoclinic phase, VO_2_ contains paired vanadium ions alongside gapped d‐orbitals. The band structure in this state displays a clear bandgap, described as *E*g. The electrical conductivity in the insulating phase follows the relation:

(1) $$\sigma_{\text{insulator}}\;\alpha \exp\;(-\frac{E_{g}}{2k_{B}T})$$

where *k*_*B*_ is the Boltzmann constant and *T* is temperature. During thermal, optical, or electronic excitation, these vanadium ion pairs dissociate, the bandgap collapses, and the material undergoes a rapid transition into the rutile metallic phase. This transition results in a dramatic increase of free‐charged carriers, where the conductivity rises sharply:

(2) $$\sigma_{\text{insulator}}\ll \sigma_{\text{metal}}$$

Ultrafast spectroscopy and quantum simulations have shown that this switching is among the fastest responses in the solid state. The transformation can be formalised through the evolution of the time‐dependent wavefunction:

(3) $$|\Psi (t)\rangle =\exp[-\frac{i}{\hbar }\int_{0}^{t}\hat{H}(\tau )d\tau ]|\Psi (0)\rangle$$

where $\hat{H}(\tau )d\tau$ represents the system Hamiltonian capturing both electronic and lattice degrees of freedom.

During ultrafast excitation, this transformation proceeds through tightly coupled electronic and lattice dynamics rather than a purely thermally driven structural change. Time‐resolved spectroscopy shows that an electronically driven collapse of the Mott‐like bandgap can occur within tens of femtoseconds, establishing a transient metallic or semi‐metallic state prior to full lattice rearrangement. Coherent oscillations of specific phonon modes in the 5–7 THz range reveal strong electron–phonon coupling, with subsequent lattice motion steering the crystal system toward the rutile structure on sub‐picosecond to picosecond timescales. Recent works further demonstrate that electronic and structural components of the transition can be partially decoupled, with nonthermal, field‐driven IMT pathways producing inhomogeneous metallic domains and metastable intermediate states that are central to device‐scale modulation behaviour [22, 23, 24, 25].

Under pulsed electrical or optical excitation, the IMT often proceeds in a spatially inhomogeneous and non‐equilibrium manner, where metallic and insulating domains coexist and percolative conduction paths form within the VO_2_ crystal system [26]. This domain evolution contributes to the characteristic thermal and electrical hysteresis, with loop width and threshold temperatures influenced by strain, grain size, and interfacial pinning, and can be exploited for multilevel, analogue modulation at the cost of increased complexity in achieving reproducible device states [27, 28].

Recent theoretical advances, including first‐principles calculations and tensor‐network modelling, highlight the dual role of strong electron correlation and lattice distortion in governing the IMT [29]. These approaches have revealed the existence of semi‐metallic intermediate states and nonadiabatic evolution of electronic bands. Control over this transformation characteristics, such as optimising the transition temperature, switching speed, and reversibility, depends on strategies like dopant selection, strain engineering, and external field application. Achieving these design objectives requires solving a complex, multidimensional materials challenge, particularly for scalable deployment in devices and adaptive electromagnetic systems.

### 2.2 Tuneable Modulation Across the Spectrum

Tuneable modulation of electromagnetic properties is one of the defining advantages of vanadium dioxide's IMT for real‐world security and communication systems. During the transition, VO_2_'s resistivity shifts sharply from insulating/semiconductor to conducting values. This change is accompanied by dramatic alterations in permittivity and reflectivity, which can span multiple orders of magnitude and directly influence how the material interacts with electromagnetic waves across the terahertz, infrared, and visible regions. In vanadium oxides hybrids complex permittivity, ε(ω,T)=ε′+iε″, varies rapidly as the temperature crosses the transition threshold, allowing devices to switch from transmitting to absorbing or reflecting light and microwaves with high fidelity [30, 31, 32].

This intrinsic tunability has enabled diverse device architectures and security functionalities. In photonics, VO_2_'s phase change is exploited for high‐speed optical modulators [33], shutters [34], and wavelength‐selective filters [35] capable of encoding, scrambling, or shielding sensitive data streams. In microwave and terahertz domains, engineered VO_2_ metasurfaces demonstrate reconfigurable absorption bands [36], polarisation control [37], and active radar tuneability [38] by varying the material's state in response to changes in environmental temperature, electric fields, or targeted optical excitation. The versatility of the IMT is further enhanced by adjusting dopant profiles [39, 40] and substrate interactions [41], which control the onset and recovery times, shift the transition temperature, and allow for dynamic tuning across user‐defined spectral ranges.

The mechanism by which VO_2_ interacts with electromagnetic radiation is strongly frequency dependent. In the microwave and terahertz regimes, the several‐orders‐of‐magnitude change in conductivity and complex permittivity across the IMT dominates, allowing VO_2_ films and metasurfaces to act as switchable resistive and inductive sheets that engineer impedance matching for broadband absorption and shielding [42, 43]. At mid‐infrared and near‐infrared wavelengths, large changes in the real and imaginary parts of the refractive index shift localised plasmonic and photonic resonances in nanoantennas, photonic crystals, and integrated waveguides, so modulation is governed primarily by resonance tuning rather than simple sheet resistance [44, 45, 46]. In the visible and near‐UV, more modest index changes still impact interference in multilayer stacks, enabling thermochromic smart coatings where VO_2_'s phase transition modulates transmittance and solar gain without relying on deep structural redesign [47, 48, 49].

For practical electromagnetic systems, the efficiency of this tuneable modulation must be evaluated in terms of the energy required to trigger a given change in optical or electrical response. Ultrafast optical studies report that absorbed fluences on the order of a few mJ cm^−2^ are sufficient to drive a long‐lived metallic state and achieve multidecibel changes in transmission or absorption, while high‐field terahertz experiments demonstrate non‐thermal IMT at fields of approximately 10–15 MV cm^−1^ [50]. In device‐relevant metasurfaces, hybrid electrical–optical driving substantially reduces the optical threshold, with pre‐biased VO_2_ elements achieving strong THz modulation at optical intensities orders of magnitude lower than purely optically driven structures. These thresholds depend sensitively on film thickness, nanostructure geometry, and interfacial thermal design, which together determine how efficiently input energy is confined to the active VO_2_ volume and dissipated during recovery [24, 51, 52].

### 2.3 Environmental Stability and Repeatability

Environmental stability and long‐term repeatability are key for the practical use of vanadium dioxide (VO_2_) devices in electromagnetic security, sensing, and smart window applications. Thin films and nanostructured VO_2_ are particularly susceptible to deterioration from oxidation, humidity, and thermal cycling, which can degrade the sharpness and speed of oxide transition and cause progressive loss of optical or electrical function over time [53, 54]. Without appropriate stabilisation, films may experience reduced transmittance modulation, increased resistivity drift, and shortened device lifetimes. Environmental degradation of VO_2_ arises from coupled chemical and mechanical pathways that progressively alter the IMT behaviour under operating conditions. Oxidation at free surfaces and grain boundaries drives the formation of higher vanadium oxides such as V_2_O_5_, which broadens the transition, shifts the threshold temperature, and reduces optical and electrical contrast over repeated cycling [55, 56]. Interfacial reactions at VO_2_/substrate or VO_2_/electrode contacts can create non‐switching interlayers that increase series resistance and pin metallic domains, while repeated volume and lattice changes across the IMT induce mechanical fatigue and microcracking in poorly buffered films [57]. Encapsulation with SiO_2_ or ZnO layers significantly suppresses oxygen and moisture ingress, stabilising the transition temperature and modulation depth over 10^3^–10^4^ thermal cycles and enabling long‐term performance in smart window and metasurface architectures [58, 59].

To address these limitations, encapsulation using silicon dioxide (SiO_2_) [60] or zinc oxide (ZnO) [61] layers acts as a physical barrier, minimising exposure to ambient oxygen and moisture and effectively preserving phase transition properties over extended cycling. For instance, core–shell nanostructures and multilayer hybrid architectures have shown marked improvements in sun modulation efficiency and luminous transmittance under harsh climatic conditions, such as sustained operation at 60°C and 90% humidity [62, 63]. Grain boundary engineering, implemented via controlled crystal growth and doping, further suppresses kinetics of degradation and helps maintain the phase transition threshold and repeatable switching behaviour over thousands of cycles. Hybrid heterostructures that combine VO_2_ with metals [64], oxides [65], or graphene [66] offer additional mechanical robustness and thermal stability, while supporting integration with electronic and photonic devices.

These advances in environmental stability and cycling endurance enable the deployment of VO_2_ thin films and coatings in demanding optoelectronic applications, infrastructure, security, and industrial contexts. Literature indicates that continued improvements in protective layer design, dopant selection, and film morphology are essential for effective large‐scale operation and viability of VO_2_‐based smart coatings and security platforms.

### 2.4 Contrasting Phase‐Change Materials for Electromagnetic Applications

To contextualise vanadium dioxide's place among phase‐change materials a direct comparison with established and emerging alternatives for electromagnetic security and adaptive systems is vital. While many materials exhibit phase transitions with electromagnetic consequences, the practical requirements of security applications narrow the field substantially: ultrafast switching, ambient operation, cycling endurance, environmental stability, and spectral coverage all matter critically [67].

Chalcogenide phase‐change materials, particularly Ge_2_Sb_2_Te_5_ (GST) and Ge_2_Sb_2_Se_4_Te_1_ (GSST), dominate optical memory and photonic switching applications [68, 69]. These materials undergo non‐volatile amorphous‐to‐crystalline transitions that produce large refractive index and absorption contrasts. However, GST requires crystallisation temperatures exceeding 160°C and operates with nanosecond switching speeds driven by structural rearrangement kinetics [69]. GSST offers improved visible and near‐infrared transparency with slightly faster switching and endurance approaching 10 million cycles but still operates near 150°C [68]. Both require energy‐intensive reset pulses to return to the amorphous state, which presents a non‐negligible drawback in large‐area deployments [70].

Magnetite (Fe_3_O_4_) presents a distinct mechanism through its Verwey transition at 124 K, where the material shifts from high‐temperature cubic metallic to low‐temperature monoclinic insulating state. This produces significant conductivity, and magnetic anisotropy changes useful for microwave absorption and shielding. The low transition temperature restricts Fe_3_O_4_ to cryogenic or specialised cooling environments, though laser‐induced photoexcitation can trigger partial transitions on picosecond timescales beyond equilibrium. Fe_3_O_4_ composites combined with carbon materials demonstrate robust mechanical properties and chemical stability [71, 72]. Halide perovskites (CsPbBr_3_, MAPbBr_3_) offer optoelectronic phase transitions at 120–235 K with picosecond to nanosecond switching speeds. These materials show size‐tuneable transitions and reversible structural phase changes useful for visible and near‐infrared modulation [73, 74]. Severe moisture sensitivity and low‐temperature operation restrict practical deployment, and, additionally, lead‐containing halide perovskites are not commercially viable in Europe due to the RoHS Directive, which has restricted lead in electronics since 2006 and effectively excludes these materials from advanced optoelectronic markets [75, 76].

By comparison, VO_2_'s volatile Mott transition near 68°C combines ambient‐temperature operation with femtosecond switching (26–75 fs), passive thermal recovery, and exceptional cycling endurance exceeding 100 million thermal cycles and 260 million electrical cycles [13, 77]. The electronically‐driven transition, rather than structural nucleation‐limited processes, enables orders‐of‐magnitude faster response than chalcogenides. The transition temperature can be tuned through doping, strain engineering, and nanostructuring toward room temperature or user‐defined thresholds [10]. VO_2_'s broadband response from ultraviolet through terahertz frequencies, combined with multistimuli triggering (thermal, optical, electrical, mechanical), provides unmatched versatility for adaptive electromagnetic architectures. Table 1 compares the key performance metrics across representative phase‐change materials for electromagnetic security applications.

**TABLE 1:** Performance comparison of phase‐change materials for electromagnetic security applications.

PCM	Transition type	Transition temperature, °C	Switching speed	Cycling endurance	Spectral coverage	Advantages	Limitations
VO_2_ [77, 78]	Mott insulator‐to‐metal	68, tuneable 20°C–90°C	26–75 fs	>100 M thermal, >260M electrical	UV–THz	Ultrafast, ambient operation, multi‐stimuli, passive recovery	Oxidation sensitivity, requires encapsulation
GST [69]	Amorphous‐to‐crystalline	>160°C	ns–μs	1–10 M	NIR–MIR	Non‐volatile memory, large optical contrast	High temperature, slow switching
GSST [68, 79]	Amorphous‐to‐crystalline	∼150°C	ns	∼10 M	Visible–NIR	Improved transparency, better endurance	High temperature, structure‐limited switching
Fe_3_O_4_ [72, 80]	Verwey transition	−149°C	ps (photo‐induced)	High	MW–THz	Magneto‐optical, chemically robust, multi‐functional	Cryogenic operation requires cooling or photoexcitation
Halide perovskites [73, 81]	Structural phase transition	120–235 K	ps–ns	Moderate	Visible–NIR	Optoelectronic functionality, size‐tuneable transition	Moisture instability, low‐temperature operation

From an application perspective, the contrast between volatile and nonvolatile phase‐change responses is as important as absolute speed or temperature [35, 36, 37]. Nonvolatile chalcogenides such as GST and GSST retain their programmed optical and electrical states without power, which is advantageous for functions like secure configuration memories, write‐once keys, and long‐term optical storage where state preservation after power loss is essential. In contrast, the volatile Mott transition of VO_2_ naturally relaxes back to the insulating state when the external stimulus is removed, eliminating the need for energy‐intensive reset pulses and making it intrinsically suited to continuous, low‐power, dynamically reconfigurable operations such as real‐time beamforming, adaptive absorption for jamming suppression, and channel randomisation in wireless links. These complementary characteristics suggest hybrid security architectures in which non‐volatile materials provide state retention and credential storage, while VO_2_ and related vanadium oxides supply agile, on‐demand modulation at the physical layer [6, 15, 17].

## 3 Synthesis and Processing

### 3.1 Physical, Chemical, Hydrothermal, and Solution Syntheses

Developing VO_2_ for practical electromagnetic security devices requires synthesis methods that deliver phase‐pure, highly crystalline material on a scale suitable for advanced optics, electronics, and coating applications. The scalability, industrial compatibility, and controllability of the synthesis route have a direct impact on the performance and reliability of VO_2_‐based systems [8].

Physical vapour deposition (PVD) methods, including pulsed laser deposition, thermal evaporation, and sputtering, are widely adopted for fabricating high‐quality thin films [82]. In PVD, vanadium‐containing targets are ablated or sputtered in an inert or reactive background at reduced pressure, followed by condensation onto heated substrates. This approach offers precise control over thickness, stoichiometry, and grain structure. Sputtering, in particular, is often selected for industrial‐scale production because it enables homogenous, reproducible films across large surface areas such as glass, silicon, quartz, and sapphire. PVD allows tuning of the IMT temperature and optical performance by adjusting oxygen partial pressure [83], substrate orientation [84], and post‐deposition annealing conditions [85]. These parameters can significantly alter the crystallinity, grain size, and phase purity, resulting in either monoclinic VO_2_(M) or other undesirable polymorphs that have drastically different transition behaviour.

**FIGURE 2:**
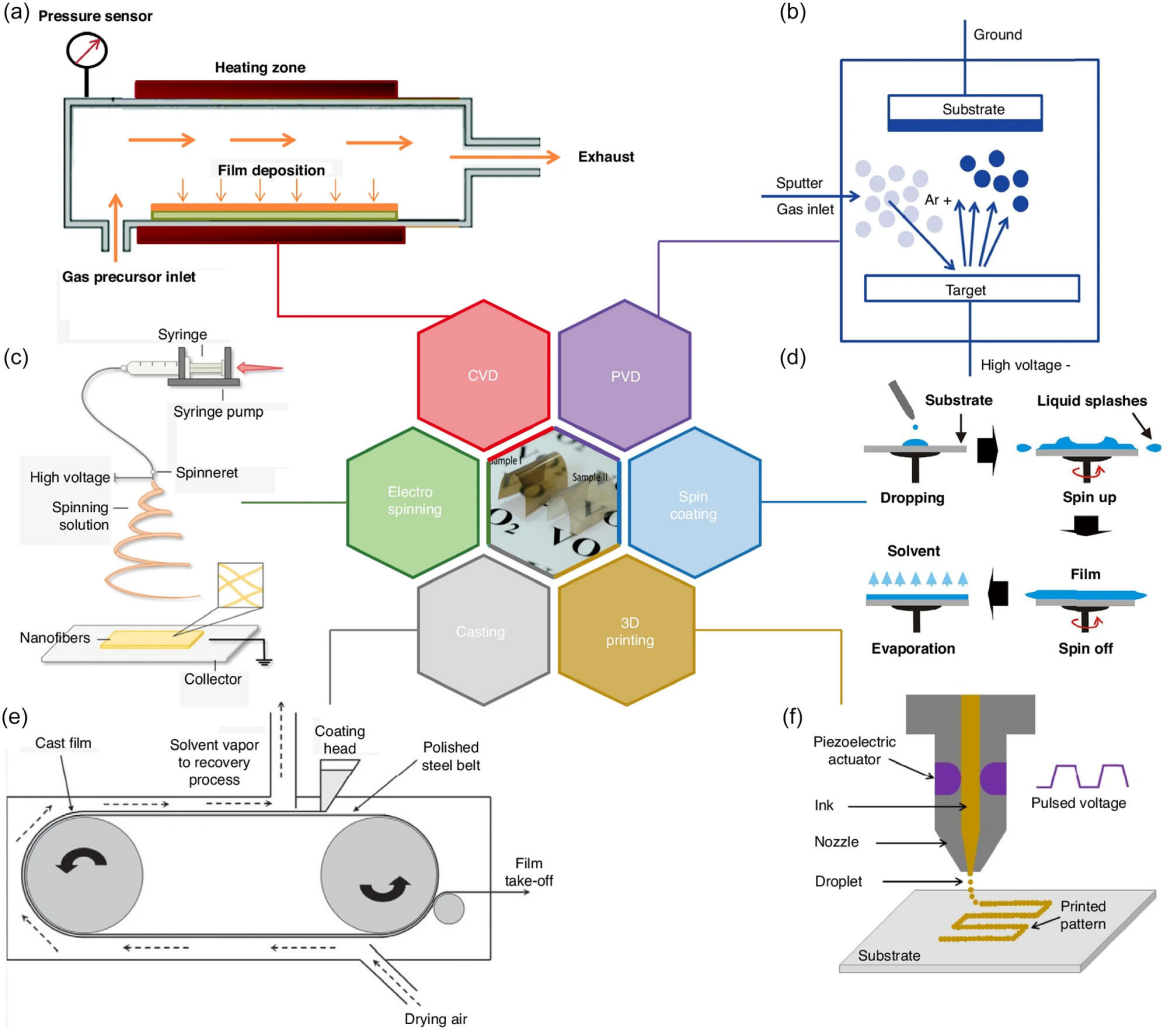
Some fabrication methods for thermochromic VO_2_ thin films. (a) Chemical vapour deposition (CVD) (b) Physical vapour deposition (PVD). (c) Electrospinning. (d) Spin coating. (e) Film casting. (f) 3D printing. Reproduced (Adapted) under the terms of the Creative Commons CC‐BY licence [61]. Copyright 2024, Cancheng Jiang et al.

Chemical vapour deposition (CVD) is another versatile route, permitting uniform and conformal growth of VO_2_ films over large and complex surfaces [86]. Both atmospheric pressure and metal‐organic CVD (MOCVD) have been demonstrated for VO_2_ [87]. Careful selection of vanadium precursors and fine control of reactor conditions, including gas flow, temperature, and oxidant ratios, are critical for ensuring functional VO_2_ phase and high purity. MOCVD, in particular, enables wafer‐scale deposition at growth rates of 14–35 nm/min for VO_2_ films with thickness uniformity < 1.2% across 75 mm substrates, and is compatible with device‐level patterning strategies, supporting integration into multilayer and multiband metamaterial architectures [88]. Figure 2 outlines some of the methods used to fabricate thermochromic VO_2_ thin films.

Hydrothermal synthesis enables the low‐temperature formation of VO_2_ nanostructures, including nanowires, nanosheets, and microcrystals. By engineering the precursor chemistry, reaction pH, temperature, and duration, hydrothermal reactions yield gram to kilogram scale crystalline products with tailored morphologies and aspect ratios [89, 90, 91]. These techniques often use safe, environmentally friendly reagents such as oxalic acid, offering a scalable and lower‐energy alternative to solid‐state synthesis. Hydrothermal approaches can also preserve desirable morphologies during phase transitions, as in the transformation from VO_2_(B) to monoclinic VO_2_(M) with post‐synthesis annealing and support the fabrication of hierarchical or composite materials for enhanced functional properties.

Beyond conventional hydrothermal and sol–gel routes, a growing class of ambient‐pressure, solution‐processed syntheses directly addresses the scalability and substrate‐compatibility limitations of high‐temperature, vacuum‐based processing. Benchtop approaches that produce VO_2_(M) nanoparticles and VO_2_‐based inks enable inkjet‐printed films and demonstrate large‐area smart windows without autoclaves or high‐temperature treatment of the final substrate. Related strategies combine VO_2_(M) nanoparticles with SiO_2_ shells to form VO_2_: SiO_2_ powders and films, where the silica simultaneously improves thermochromic performance and protects against grain growth and oxidation during annealing. In this context, ambient‐pressure core–shell routes that synthesise *VO_x_·*nH_2_O and VO_2−*x*_ nanoparticles and subsequently encapsulate them via Stöber‐type silica growth provide scalable suspensions that leverage both intrinsic strain from oxygen deficiency and extrinsic strain from the silica shell to tune the IMT toward near‐room temperature, while remaining compatible with room‐temperature coating of glass, polymers, and fabrics. Collectively, these nonstandard solution processes complement sputtered and MOCVD films by offering retrofit‐friendly, mechanically flexible VO_2_ coatings that are intrinsically suited to large‐area electromagnetic and smart‐window applications.

Recent advances in vanadium oxide synthesis extend these ambient and hydrothermal strategies by exploiting hybrid and unconventional approaches to optimise phase‐transition properties at the nanoscale [92, 93, 94, 95]. One notable direction involves the fabrication of core–shell and heterostructured nanomaterials, where encapsulation and compositional gradients are used primarily to engineer strain, optical response, and damage thresholds rather than to enable basic scalability. For instance, VO_2_ nanoparticles encapsulated with a SiO_2_ shell harness the differential thermal expansion between core and shell to induce controllable internal strain, enabling significant reduction of the IMT temperature toward room temperature and supporting the design of high‐performance photonic modulators and laser components. Other innovative routes include bio‐assisted synthesis, combinatorial deposition, and strain‐engineered growth on 2D materials, which collectively allow fine control over stoichiometry, defect density, and lattice distortion for device‐grade thin films and nanostructures [92]. Related work demonstrates that VO_2_/V_2_O_5_ core–shell heterostructures, which can be formed naturally in ambient conditions, deliver broadened infrared and nonlinear optical responses, exceptional damage thresholds, and unique saturable absorption profiles that are challenging to achieve in monolithic VO_2_ [93]. These hybrid nanostructures now underpin the design of high‐performance photonic modulators and laser components.

Other innovative routes include green, bio‐assisted synthesis. For instance, biogenic methods utilising Shewanella bacteria achieve VO_2_ nanoparticle formation at low temperature and ambient pressure, eliminating harsh chemical reductants and minimising environmental impact [94]. These biosynthesized VO_2_ nanomaterials exhibit well‐defined phase transition temperatures and offer additional opportunities for surface engineering through vesicle‐mediated assembly and natural membrane encapsulation. Table 2 summarises how sputtering, MOCVD, conventional hydrothermal/solution routes, and emerging ambient core–shell and ink‐based processes occupy complementary niches in terms of production scale, platform integration, and durability requirements for VO_2_‐based electromagnetic security devices.

**TABLE 2:** Comparison of the industrial viability of major vanadium dioxide synthesis methods.

Synthesis route	Typical outputs and scale	Integration strengths	Durability and encapsulation considerations
PVD/sputtering	Large‐area VO_2_ films on glass, Si, quartz and sapphire with good thickness uniformity.	Widely used for architectural coatings and RF substrates; compatible with back‐end processing under controlled thermal budget and contamination.	Dense films but sensitive to off‐stoichiometry; stability improved by SiO_2_/ZnO capping and grain‐boundary engineering.
MOCVD	Conformal VO_2_ films on planar and 3D wafer‐scale substrates with uniformity < ∼1.2% at 14–35 nm min^−1^ growth rates.	Well suited to integrated photonics and metasurfaces with good interface and composition control within CMOS‐compatible temperatures.	Requires encapsulation to suppress oxidation and humidity; process conditions set defect density and cycling endurance.
Hydrothermal/conventional solution	Gram–kilogram VO_2_ nanostructures (nanowires, plates, powders) later converted into films or composites.	Supports printing, spraying, or casting on diverse substrates for low‐cost, large‐area smart coatings.	Film robustness dominated by binders, porosity and added barriers; extra encapsulation needed to match dense vacuum‐grown films.
Ambient benchtop core–shell/ink routes	Ambient‐pressure VO_2_(M) and core–shell nanoparticles (e.g. VO_2_: SiO_2_, VO_2−*x*_ from *VO_x_·*nH_2_O) formulated as inks without autoclaves.	Enable inkjet, glazing and screen‐printed VO_2_ coatings on glass, polymers and fabrics at or near room temperature for retrofit and flexible devices.	Silica shells and polymer matrices provide strain engineering and environmental protection, tuning IMT toward near‐room temperature while enhancing cycling stability; long‐term behaviour set by shell and matrix integrity.

Combinatorial and strain engineering approaches also play an expanding role. Wafer‐scale combinatorial deposition techniques, such as those that spatially vary oxygen partial pressure or alloy composition across a substrate, allow rapid mapping and optimisation of stoichiometry, phase purity, and IMT properties for electronic‐grade thin films. Strain‐engineered growth, for example by depositing VO_2_ on flexible 2D materials such as hexagonal boron nitride (hBN), yields ultrathin films with preserved transition characteristics and minimised defect‐induced degradation [95]. These strain‐free or strain‐tailored films can be tuned for thickness dependence, reversibility, and device miniaturisation, all of which are difficult to achieve with conventional bulk processing.

## 4 Metasurface and Terahertz Security Applications

### 4.1 Broadband Absorbers and Tuneable Shielding

VO_2_‐based terahertz (THz) metasurface absorbers are demonstrating unprecedented performance in bandwidth, tunability, and device thickness. A recent design achieved a broadband absorption of 6.35 THz, with absorptance greater than 90% from 2.82 to 9.17 THz simply by tuning the VO_2_ conductivity between 200 and 200,000 S/m [14]. The absorber is composed of a single‐layer, patterned VO_2_ resonator placed on a quartz dielectric spacer, and backed by a metallic ground plane. Simulations and experiments showed polarisation‐insensitive perfect absorption and wide‐angle stability, with tunability of the absorption peak from 2% to 100%. The physical mechanisms were modelled using impedance matching theory, electric field and surface current distributions, and parametric changes in layer thickness and periodicity. Devices like this outperform prior structures both by eliminating stray transmission and by offering seamless integration of stealth, THz imaging, and secure communication functionalities.

**FIGURE 3:**
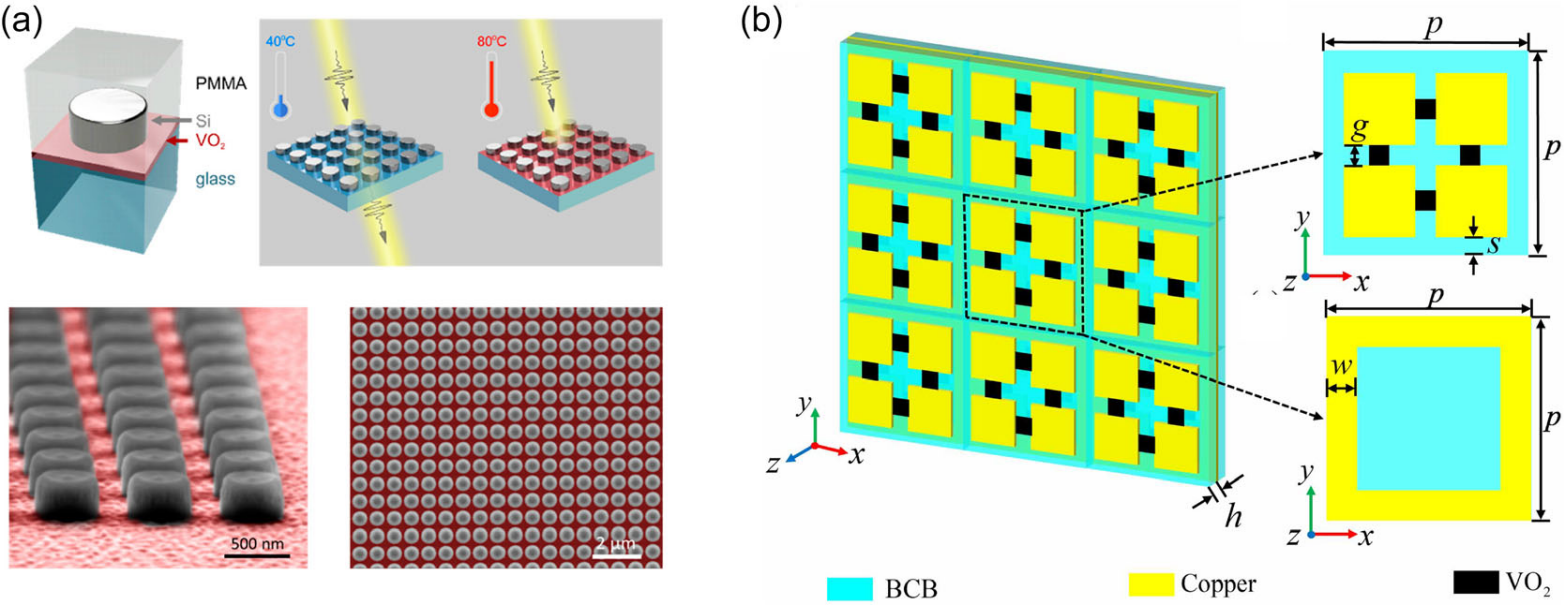
(a) Design concept for a tuneable dielectric metasurface: a silicon meta‐atom is embedded in a glass or PMMA matrix with a buried 25 nm VO_2_ layer; the Si disk is 240 nm high, 255 nm in radius, and arranged in a square lattice with 700 nm period, topped with a 500 nm PMMA layer. Reproduced (Adapted) with permission [98]. Copyright 2021, American Chemical Society (ACS). (b) Schematic of the proposed VO_2_‐based metasurface filter for THz energy‐selective surface applications. Reproduced (Adapted) with permission [99]. Copyright 2024, IOP publishing.

In other works, a patterned VO_2_ metasurface absorber incorporating a low‐loss MF_2_ dielectric and gold ground plane achieved an absorptance of 98.15% across a 5.38 THz [96] bandwidth, from 5.72 to 11.11 THz, even at incident angles deviating significantly from normal [3]. These absorbers are suitable for physical layer security since wide‐angle and polarisation‐insensitive operation prevents information leakage and eavesdropping attempts that might exploit incident field geometry [97]. Figure 3 shows advanced VO_2_‐based metasurface designs that leverage the phase‐change properties of vanadium dioxide to create tuneable, reconfigurable optical and terahertz devices.

### 4.2 Beam Steering and Programmable Intelligent Reflecting Surfaces (IRS)

**FIGURE 4:**
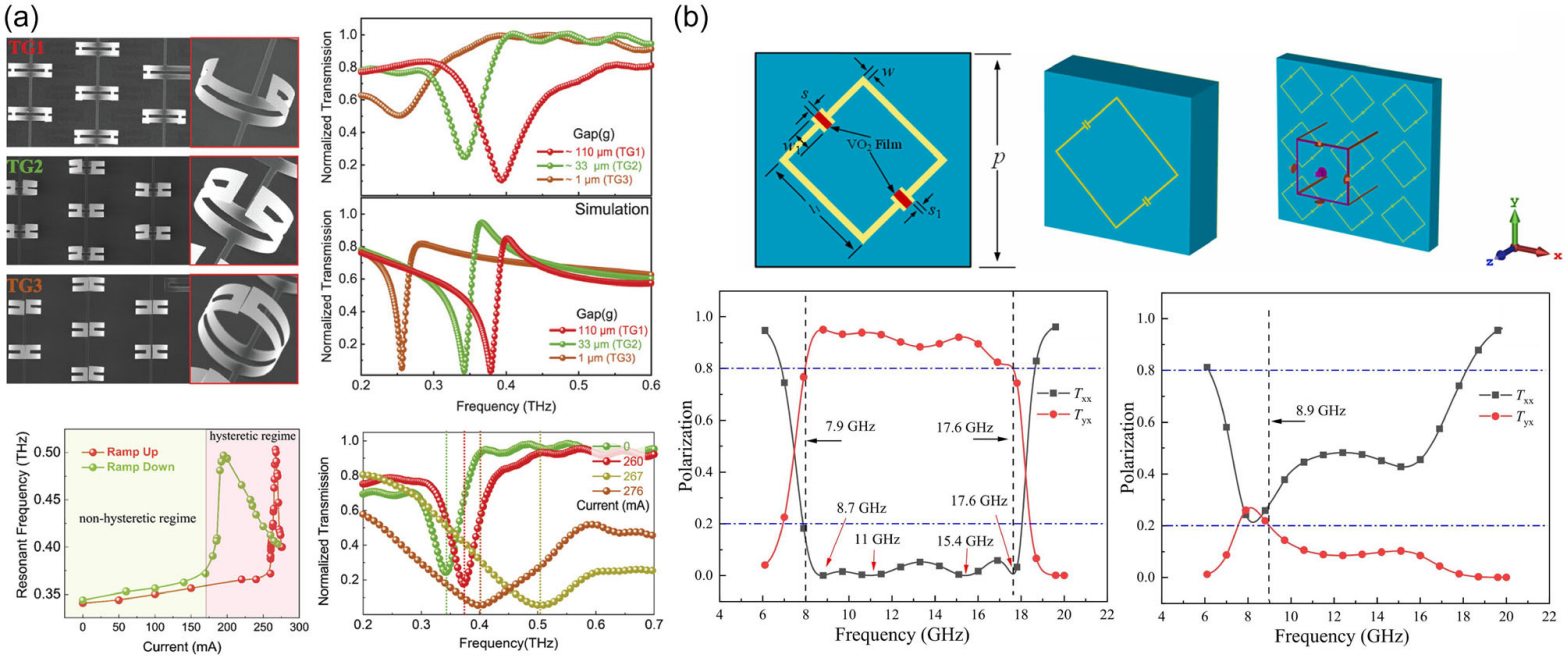
(a) VO_2_ resonator design with bias lines and measured transmission of shunt switches. Reproduced (Adapted) with permission [106]. Copyright 2024, John Wiley and Sons. (b) VO_2_‐based polarisation conversion metasurface and polarisation ratios below and above the phase transition temperature. Reproduced (Adapted) with permission [107]. Copyright 2022, Elsevier.

VO_2_ metasurfaces also support precise, real‐time beam steering across THz, mmWave, and microwave frequencies. For example, a programmable metasurface operating at 100 GHz used VO_2_ as the switching substrate to achieve up to 44° beam deflection in both horizontal and vertical directions. This performance approaches traditional electronically‐steered phased arrays that typically achieve ±50°–70° coverage, while offering significantly faster switching speeds and eliminating the complex feeding networks required by conventional architectures. Unlike mechanical beam‐steering approaches that operate on second timescales, VO_2_ metasurfaces enable sub‐millisecond electronic control with solid‐state reliability [100, 101, 102]. The phase transition, controlled by temperature or electrical biasing, altered the refractive index from 2.6 to 36 and extinction coefficient from near zero to 16 at 100 GHz, enabling sharp and reversible steering [96]. Additionally, when combining electrical and optical stimulation applied voltage can drop the switching threshold for optical beam steering, making programmable phase control feasible with moderate currents or optical intensities [103]. Advanced coding metasurfaces with VO_2_ digital elements have been used to dynamically configure both the spatial phase profile and beam topology, allowing for secure, adaptive link routing in wireless communications and imaging systems [40, 100, 104, 105]. Figure 4 shows VO_2_‐based resonator and metasurface structures with their corresponding transmission and polarisation responses across the phase transition.

### 4.3 Secure Optical Switching and Data Scrambling

VO_2_‐based phase change materials are an excellent contender for developing ultrafast and reprogrammable optical switches, modulators, and memory elements, combining scalability with true device‐level tunability. The femtosecond to picosecond timescales IMT of VO_2_ hybrids powers ultrafast, nonmechanical modulation in both amplitude and phase. To this end, tuneable optical switches that utilise plasmonic structure of Aluminium nanoarrays coupled with a VO_2_ thin film can achieve a modulation depth of 99.4% and an extinction ratio exceeding −22 dB, with the switching time of the VO_2_ phase transition approaching 150 fs [108]. It is noteworthy that the phase transition temperature of VO_2_ is only 68°C, far lower than that of conventional phase change materials like Ge_2_Sb_2_Te_5_ (typically above 150°C), which translates to lower operating energy and improved device integration for mid‐infrared applications [109].

In the near‐infrared and telecommunication regimes, hybrid integrated photonic circuits leverage the large and broadband refractive index change of VO_2_ to achieve high‐contrast, compact amplitude and phase modulation [110]. Hybrid Si–VO_2_ waveguide structures, where the active VO_2_ patch is triggered thermally or electrically, demonstrate absorption modulation as high as 4 dB/μm and intracavity phase modulation approaching π/5 per micron of active length, allowing dynamic, on‐chip reconfiguration of optical resonance and up to 7 dB optical modulation in microring resonators. Integrating these volatile VO_2_ elements into practical encryption and photonic platforms raises distinct system‐level considerations. On silicon and silicon‐nitride photonics, VO_2_ can be deposited by sputtering or MOCVD within back‐end‐of‐line thermal budgets, but requires careful control of stress, interdiffusion, and contamination through buffer and barrier layers to avoid degrading both the IMT and underlying circuitry [34, 41]. Because VO_2_ does not retain its state without bias, nonvolatile memory requirements in secure systems such as long‐term key storage must be met by complementary technologies (for example GST‐based photonic memory, RRAM, or MRAM), while VO_2_ is best reserved for stateless physical‐layer functions that benefit from automatic reset, including transient beam shaping, channel randomisation, and rapid erasure of optical paths upon power loss [67, 69, 79, 111]. These hybrid micro/nano structured circuits have broadband responses that can be programmed or switched at rates exceeding 100 GHz, making them suitable for high‐speed encryption, routing, and photonic memory in secure networks [112, 113].

Furthermore, the use of VO_2_ metasurfaces with engineered nanoantennas for independent and dual‐function amplitude and phase control, with continuous modulation demonstrated over 120° phase and nearly 10 dB amplitude in repeatable cycles without degradation, enabling access to intermediate states [113]. These intermediate states are useful for robust, dynamic optical modulation and show potential for advanced optical encryption or beam‐steering, including for LIDAR or holographic displays [114]. These intermediate, partially switched states correspond microscopically to mixed‐phase domain configurations within the VO_2_ layer and are accessed along the hysteresis loop rather than at its binary end points [115]. While they enable continuous tuning of amplitude and phase for advanced beam shaping and encryption [114], they also require calibration and closed‐loop control, since environmental drift or device ageing can shift the effective thresholds that map bias conditions to specific electromagnetic responses. Nonetheless, VO_2_‐based integrated photonic devices uniquely combine volatile, reconfigurable switching with fast, scalable, and repeatable operation, making them ideal for reconfigurable and physically secure optical hardware [116].

## 5 Threat Mitigation Mechanisms and Security Architectures

The security functions enabled by VO_2_, such as absorption, beam steering, and dynamic modulation, have greater utility when coupled within broader architectures that combine sensing, control logic, and communication. Rather than a static mapping between specific threats and fixed countermeasures, VO_2_‐based surfaces can realise proactive, intelligent physical‐layer security by continuously adapting their electromagnetic response in time, frequency, and space. In this context, tuneable absorbers, programmable IRS, and tamper‐responsive devices serve as reconfigurable building blocks for higher‐level capabilities including adaptive jamming shields, channel‐randomising links, and self‐erasing hardware platforms.

### 5.1 Jamming and Interference Protection

VO_2_‐based metasurface devices provide adaptive, real‐time protection against deliberate jamming and unintentional interference. In this context, the broadband absorbers and beam‐steering metasurfaces introduced in Sections 4.1 and 4.2 act as reconfigurable front‐ends within an adaptive jamming‐protection architecture, where VO_2_'s state is continuously tuned in response to sensed interference to suppress hostile signals while preserving desired links. In recent research, a C4‐symmetry‐broken VO_2_ metasurface was designed to create dynamically tuneable transmission characteristics that can be rapidly reconfigured through thermal activation or optical control. By switching the local conductivity of VO_2_ from insulating (about 200 S/m) to metallic (up to 2 × 10^5^ S/m), the device demonstrated drastic changes in transmittance, reflectivity, and absorptivity modifying the electromagnetic radiation within sub‐millisecond time frames [19, 117]. During high‐temperature operation, the metasurface supports new coupling modes and modified electromagnetic‐induced transparency windows, which allow the device to absorb, redirect, or scatter targeted jamming signals with high effectiveness. These tuneable properties enable the deployment of adaptive channel blockers, spectral filters, and dynamic noise‐shaping capabilities that are critical for preventing high‐intensity or frequency‐agile jamming attacks in secure THz and wireless networks [19, 118, 119, 120]. Rather than functioning as static absorbers, these VO_2_‐based metasurfaces illustrate how tuneable loss, phase, and polarisation control can be composed into closed‐loop jamming shields in which electromagnetic functions are embedded in a broader sensing and decision stack.

### 5.2 Eavesdropping and Channel Randomisation

Advances in VO_2_ phase‐change metasurfaces have enabled on‐demand physical channel randomisation, which is vital for physical‐layer security. The abrupt change in VO_2_'s complex refractive index and permittivity at the IMT causes the physical properties of metasurfaces and large reflecting surfaces to vary between measurements, a property that is directly exploited to generate encryption keys based on dynamic channel state information [111, 121]. Here, VO_2_‐programmable metasurfaces operate as IRS that implement channel‐randomising wireless architectures, using the beam‐steering and modulation capabilities detailed in Sections 4.2 and 4.3 to deliberately shape the propagation environment and embed security into the physical layer. Using multifunctional bias‐encoded VO_2_ metasurfaces demonstrated that encoding states can be rapidly cycled among multiple discrete and continuous values, producing programmable wavefronts and arbitrary beam patterns at THz frequencies [103, 122]. Such architectures are ideally suited for wireless authentication, key exchange, and secure communications, where traditional eavesdropping fails due to the complexity and rapidity of channel evolution [123]. These systems demonstrate that VO_2_'s rapid, reversible phase transition enables security architectures in which the physical channel itself becomes a dynamic cryptographic resource, rather than a passive medium that must be protected solely by higher‐layer protocols.

### 5.3 Hardware‐Level Tamper Resistance

**FIGURE 5:**
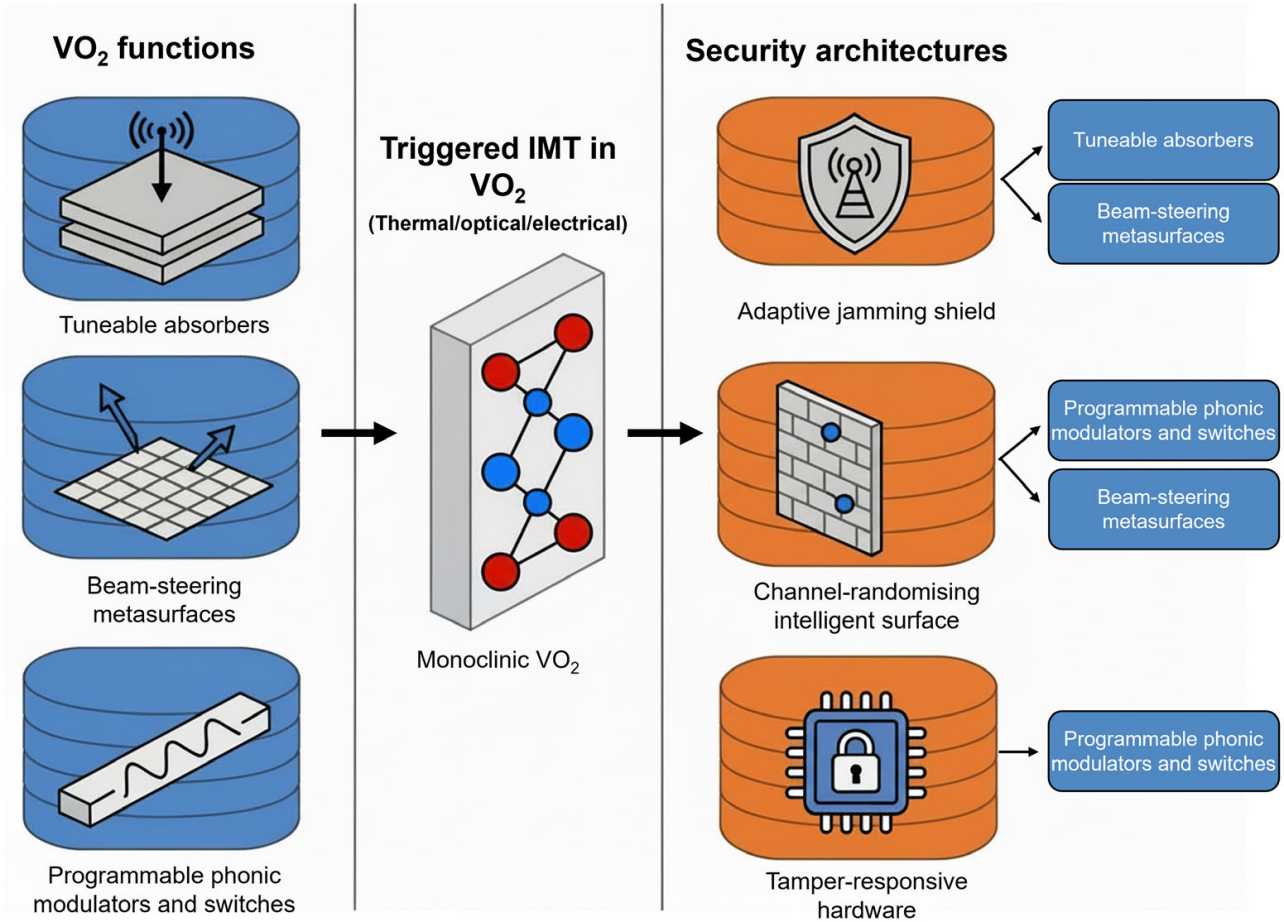
Monoclinic VO_2_ provides tuneable absorption, beam steering and photonic modulation functions that serve as building blocks (left) for higher‐level electromagnetic security architectures (right), including adaptive jamming shields, channel‐randomising intelligent reflecting surfaces and tamper‐responsive hardware platforms.

Physical integration of VO_2_ within semiconductor and photonic devices offers robust, intrinsic tamper‐resistance for memory, logic, and sensor networks. A multiphysical‐field modulated VO_2_ device produced information encryption by encoding data as “0” and “1” states, which could be reliably switched and erased by electric fields, thermal pulses, or light irradiation, with almost instantaneous transition and high device stability [124, 125, 126]. When integrated with sensors, control logic, and nonvolatile memories, such VO_2_ elements form tamper‐responsive hardware architectures in which physical stimuli trigger rapid erasure, reconfiguration, or lock‐down of critical functions leveraging the volatile IMT as a built‐in ‘self‐destruct’ or quarantine mechanism. In recent advanced functional materials demonstrations, this approach was further extended by combining VO_2_ phase‐switching with Li‐ion modulation, enabling battery‐less security triggers and ‘fail‐safe’ erasure mechanisms. Such active material integration brings anti‐tamper, and reconfigurable protection to IoT, secure computation, and edge devices, marking an evolution beyond traditional hardware security methods [124]. In Figure 5, we position monoclinic VO_2_ as a central phase‐change platform that first enables tuneable absorption, beam steering, and photonic modulation functions, which are then composed into higher‐level electromagnetic security architectures, including adaptive jamming shields, channel‐randomising IRS and tamper‐responsive hardware. In this way, VO_2_ is positioned not merely as a switchable material but as a cornerstone of hardware architectures where security emerges from coordinated material response, circuit design, and system‐level policy.

## 6 Conclusion

Recent advances position vanadium dioxide (VO_2_) as a leading material for adaptive electromagnetic modulation, physical‐layer security, and optical switching. Its broadband response, rapid tunability, and near‐room‐temperature switching distinguish VO_2_ from other phase‐change materials, meeting the requirements of smart coatings, reconfigurable sensors, and secure electromagnetic environments.

Widespread adoption depends on overcoming two persistent barriers: the lack of scalable, reproducible synthesis methods for high‐quality VO_2_ and its hybrids, and the need for proven long‐term durability in practical environments. Recent developments in synthesis that exploit strain engineering, strategic doping, and advanced encapsulation offer viable pathways toward large‐scale production and standardisation, enabling broader deployment and accelerating the field.

Future research should prioritise durability and cycling studies, with emphasis on thin films and nanostructures that remain vulnerable to oxidation, humidity, and thermal stress. Resolving these challenges will support the translation of VO_2_ technologies into physically secure, reliable electromagnetic and optical devices for mission‐critical applications. Opportunities remain in developing advanced encapsulation strategies, strengthening compositional and structural stability, and integrating VO_2_ into flexible or multifunctional platforms for extended operational lifetimes.

VO_2_'s combination of fast, broadband modulation and practical operating conditions positions it as a foundational platform for future electromagnetic security technologies. Scalable synthesis, systematic reliability testing, and robust device integration are essential for moving from laboratory demonstrations to real‐world deployment. Progress in sputtered and MOCVD films, scalable nanoparticle and ink‐based solution routes, and SiO_2_/ZnO or core–shell encapsulation strategies indicates that both large‐area manufacturability and long‐term durability of VO_2_ devices are becoming compatible with practical electromagnetic security architectures.

Establishing durability standards and best practices will enable VO_2_ to support a new generation of adaptive, robust electromagnetic systems in communications, infrastructure, and advanced sensing.

## Funding

This research was conducted by the University of Melbourne for National Intelligence Postdoctoral Grant (project number NIPG202502) and funded by the Australian Government.

## Conflicts of Interest

The authors declare no conflicts of interest.

## Data Availability

Data sharing is not applicable to this article, as no new data were created or analyzed in this study.

## References

[1] Y. I. Abdulkarim , H. N. Awl , F. F. Muhammadsharif , et al., “A Vanadium Dioxide‐Based Metamaterial with Quatrefoil and Circle Loaded Structure on Flexible Polyamide Substrate for Terahertz Applications,” Frontiers in Physics 10 (2022): 968310.

[2] H. Jiang , Y. Wang , Z. Cui , X. Zhang , Y. Zhu , and K. Zhang , “Vanadium Dioxide‐Based Terahertz Metamaterial Devices Switchable between Transmission and Absorption,” Micromachines 13, no. 5 (2022): 715. 35630181 10.3390/mi13050715 PMC9145035

[3] Y. Wang , Y. Chen , F. Liu , et al., “Vanadium Dioxide Enabled Polarization Insensitive Tunable Broadband Terahertz Metamaterial Absorber,” Scientific Reports 15, no. 1 (2025): 10140. 40128582 10.1038/s41598-025-94645-3 PMC11933408

[4] C. Zhou , Y. Feng , T. Wang , W. Jin , Y. Chen , and Y. Lu , “In Design and Study of Vanadium Dioxide Materials for Laser Protection, Sixth International Conference on Optoelectronic Science and Materials (ICOSM),” SPIE 2024 (2024): 159–164.

[5] B. Li and Y.‐S. Lin , “Tunable Terahertz Perfect Absorber Using Vanadium Dioxide‐Based Metamaterial for Sensing Applications,” Journal of Alloys and Compounds 983 (2024): 173922.

[6] J. Yoon , W.‐K. Hong , Y. Kim , and S.‐Y. Park , “Nanostructured Vanadium Dioxide Materials for Optical Sensing Applications,” Sensors 23, no. 15 (2023): 6715. 37571499 10.3390/s23156715 PMC10422301

[7] Y. Zhang , W. Xiong , W. Chen , and Y. Zheng , “Recent Progress on Vanadium Dioxide Nanostructures and Devices: Fabrication, Properties, Applications and Perspectives,” Nanomaterials 11, no. 2 (2021): 338. 33525597 10.3390/nano11020338 PMC7911400

[8] D. Sun , C. W. Kwon , G. Baure , et al., “The Relationship between Nanoscale Structure and Electrochemical Properties of Vanadium Oxide Nanorolls,” Advanced Functional Materials 14, no. 12 (2004): 1197–1204.

[9] K. Chen , W. Song , Z. Li , et al., “Chalcogenide Phase‐Change Material Advances Programmable Terahertz Metamaterials: A Non‐Volatile Perspective for Reconfigurable Intelligent Surfaces,” Nanophotonics 13, no. 12 (2024): 2101–2105. 39634494 10.1515/nanoph-2023-0645 PMC11501539

[10] J. Cui , Q. Jiang , N. Wang , and S. Liang , “Regulating the Phase Transition Temperature of VO2 Films via the Combination of Doping and Strain Methods,” AIP Advances 13, no. 5 (2023).

[11] S. W. Schmid , L. Pósa , T. N. Torok , et al., “Picosecond Femtojoule Resistive Switching in Nanoscale VO2 Memristors,” ACS Nano 18, no. 33 (2024): 21966–21974. 39115225 10.1021/acsnano.4c03840 PMC11342367

[12] I. Vassalini , I. Alessandri , and D. de Ceglia , “Stimuli‐Responsive Phase Change Materials: Optical and Optoelectronic Applications,” Materials 14, no. 12 (2021): 3396. 34205233 10.3390/ma14123396 PMC8233899

[13] S. Chen , M. Lust , A. Roo , and N. Ghalichechian , “Reliability of VO Textsubscript 2‐Based mmWave Switches under 100 million Thermal Cycles,” IEEE Transactions on Device and Materials Reliability 23, no. 2 (2023): 241–248.

[14] G. Yang , F. Yan , X. Du , et al., “Tunable Broadband Terahertz Metamaterial Absorber Based on Vanadium Dioxide,” Aip Advances 12 (2022): 4.

[15] H. Kayan , M. Nunes , O. Rana , P. Burnap , and C. Perera , “Cybersecurity of Industrial Cyber‐Physical Systems: A Review,“ ACM Computing Surveys 54, no. 11 (2022): 1–35.

[16] A. Burg , A. Chattopadhyay , and K.‐Y. Lam , “Wireless Communication and Security Issues for Cyber–physical Systems and the Internet‐of‐Things,” Proceedings of the IEEE 106, no. 1 (2017): 38–60.

[17] L. Kong , W. Li , T. Zhang , et al., “Wireless Technologies in Flexible and Wearable Sensing: From Materials Design, System Integration to Applications,” Advanced Materials 36, no. 27 (2024): 2400333. 10.1002/adma.202400333 38652082

[18] Q. Chen , Y. Cheng , W. Min , Y. Xiao , and Z. Xia , “A Composite Energy‐Selective Surface Based on Diode‐Induced VO2 Conduction for the Applications of Adaptive Electromagnetic Protection,” Microwave and Optical Technology Letters 66, no. 1 (2024): e33895.

[19] D. Wang , B. Cai , L. Yang , et al., “Transmission/Reflection Mode Switchable Ultra‐Broadband Terahertz Vanadium Dioxide (VO2) Metasurface Filter for Electromagnetic Shielding Application,” Surfaces and Interfaces 49 (2024): 104403.

[20] M. F. Becker , A. B. Buckman , R. M. Walser , T. Lépine , P. Georges , and A. Brun , “Femtosecond Laser Excitation of the Semiconductor‐Metal Phase Transition in VO2,” Applied Physics Letters 65, no. 12 (1994): 1507–1509.

[21] K. A. Hallman , K. J. Miller , A. Baydin , S. M. Weiss , and R. F. Haglund , “Sub‐Picosecond Response Time of a Hybrid VO2: Silicon Waveguide at 1550 nm,” Advanced Optical Materials 9, no. 4 (2021): 2001721.

[22] D. Wegkamp and J. Stähler , “Ultrafast Dynamics during the Photoinduced Phase Transition in VO2,” Progress in Surface Science 90, no. 4 (2015): 464–502.

[23] M. F. Jager , C. Ott , P. M. Kraus , et al., “Tracking the Insulator‐to‐Metal Phase Transition in VO2 with Few‐Femtosecond Extreme UV Transient Absorption Spectroscopy,” Proceedings of the National Academy of Sciences 114, no. 36 (2017): 9558–9563. 10.1073/pnas.1707602114 PMC5594684 28827356

[24] A. Pashkin , C. Kubler , and H. Ehke et al., “Ultrafast Insulator‐Metal Phase Transition in VO. 2 Studied by Multiterahertz Spectroscopy,” Physical Review B—Condensed Matter and Materials Physics 83, no. 19 (2011): 195120.

[25] J. Xu , D. Chen , and S. Meng , “Decoupled Ultrafast Electronic and Structural Phase Transitions in Photoexcited Monoclinic VO2,” Science Advances 8, no. 44 (2022): eadd2392. 36332024 10.1126/sciadv.add2392 PMC9635820

[26] B. Rajeswaran and A. Umarji , “Phase Evolution and Infrared Transmittance in Monophasic VO2 Synthesized by a Rapid Non‐Equilibrium Process,” Materials Chemistry and Physics 190 (2017): 219–229.

[27] F. Dumas‐Bouchiat , M. Gaudin , I. A. Zapata , and C. Champeaux , “VO2 Thin Films: Various Microstructures for Hysteresis Manipulations,” Vacuum 227 (2024): 113408.

[28] I. Voloshenko , F. Kuhl , B. Gompf , et al., “Microscopic Nature of the Asymmetric Hysteresis in the Insulator‐Metal Transition of VO2 Revealed by Spectroscopic Ellipsometry,” Applied Physics Letters 113 (2018): 20.

[29] L. Zhang , U. Bhattacharya , M. Recasens , et al., “Tensor Network Study of the Light‐Induced Phase Transitions in Vanadium Dioxide,” Npj Quantum Materials 10, no. 1 (2025): 32.

[30] J. Ramsey , S. Lee , W. Disharoon , D. West , and N. Ghalichechian , “Low‐Loss Vanadium Dioxide‐Enabled mmWave Tunable Reflective Electromagnetic Surface with Complementary Unit Cells for Wave Manipulation,” Journal of Applied Physics 135, no. 21 (2024).

[31] Z. G. Ban , Y. Shi , N. Q. Huang , et al., “Modeling Terahertz Properties of Vanadium Dioxide by Ab Initio Computational Scheme and Its Experimental Verification,” Physical Review Applied 18, no. 6 (2022): 064095.

[32] L. M. Wheeler , T. L. Phan , M. A. Smeaton , et al., “Tuning Optical and Electrical Properties of Vanadium Oxide with Topochemical Reduction and Substitutional Tin,” Chemistry of Materials 36, no. 21 (2024): 10483–10495. 39554284 10.1021/acs.chemmater.4c01557 PMC11562072

[33] M. Heidari , V. Faramarzi , Z. Sharifi , et al., “A High‐Performance TE Modulator/TM‐Pass Polarizer Using Selective Mode Shaping in a VO2‐Based Side‐Polished Fiber,” Nanophotonics 10, no. 13 (2021): 3451–3463.

[34] J. Figueroa , H. Dsouza , J. Pastrana , et al., “VO2‐Based Micro‐Electro‐Mechanical Tunable Optical Shutter and Modulator,” Optics Express 29, no. 16 (2021): 25242–25253. 34614858 10.1364/OE.428165

[35] D. Chauhan , Z. Sbeah , R. Adhikari , M. S. Thakur , S. H. Chang , and R. P. Dwivedi , “Theoretical Analysis of VO2 Filled Double Rectangular Cavity‐Based Coupled Resonators for Plasmonic Active Switch/Modulator and Band Pass Filter Applications,” Optical Materials 125 (2022): 112078.

[36] A. Tripathi , J. John , S. Kruk , et al., “Tunable Mie‐Resonant Dielectric Metasurfaces Based on VO2 Phase‐Transition Materials,” ACS Photonics 8, no. 4 (2021): 1206–1213.

[37] Y. He , B. Cai , L. Wu , et al., “Tunable VO2 Metasurface for Reflective Terahertz Linear and Circular Polarization Wavefront Manipulation at Two Frequencies Independently,” Physica B: Condensed Matter 681 (2024): 415848.

[38] H. Liu , X. Sun , L. Liu , G. Qin , and T. Li , “Ultra‐Wideband Switchable Multifunctional Terahertz Hypersurfaces Based on Graphene and VO2,” Physica Scripta 100, no. 8 (2025): 085513.

[39] Y. Wu , L. Fan , Q. Liu , et al., “Decoupling the Lattice Distortion and Charge Doping Effects on the Phase Transition Behavior of VO2 by Titanium (Ti4+) Doping,” Scientific Reports 5, no. 1 (2015): 9328. 25950809 10.1038/srep09328 PMC4423444

[40] Y. Gao , C. Cao , L. Dai , et al., “ Phase and Shape Controlled VO. 2 Nanostructures by Antimony Doping,” Energy & Environmental Science 5 (2012):8708–8715.

[41] A. Atul , M. Ahmadi , P. Koutsogiannis , H. Zhang , and B. J. Kooi , “Strong Substrate Influence on Atomic Structure and Properties of Epitaxial VO2 Thin Films,” Advanced Materials Interfaces 11, no. 3 (2024): 2300639.

[42] H. Wei , J. Gu , T. Zhao , et al., “Tunable VO2 Cavity Enables Multispectral Manipulation From Visible to Microwave Frequencies,” Light: Science & Applications 13, no. 1 (2024): 54. 10.1038/s41377-024-01400-w PMC10879493 38378739

[43] J. S. Schalch , Y. Chi , Y. He , et al., “Broadband Electrically Tunable VO2‐Metamaterial Terahertz Switch with Suppressed Reflection,” Microwave and Optical Technology Letters 62, no. 8 (2020): 2782–2790.

[44] M. J. Dicken , K. Aydin , I. M. Pryce , et al., “Frequency Tunable Near‐Infrared Metamaterials Based on VO2 Phase Transition,” Optics Express 17, no. 20 (2009): 18330–18339. 19907624 10.1364/OE.17.018330

[45] M. M. Qazilbash , M. Brehm , B.‐G. Chae , et al., “Mott Transition in VO2 Revealed by Infrared Spectroscopy and Nano‐Imaging,” Science (new York, N.y.) 318, no. 5857 (2007): 1750–1753. 18079396 10.1126/science.1150124

[46] S. Liang , F. Xu , W. Li , et al., “Tunable Smart Mid Infrared Thermal Control Emitter Based on Phase Change Material VO2 Thin Film,” Applied Thermal Engineering 232 (2023): 121074.

[47] G. Li , D. Xie , Z. Zhang , et al., “Flexible VO2 Films for in‐Sensor Computing with Ultraviolet Light,” Advanced Functional Materials 32, no. 29 (2022): 2203074.

[48] G. Li , D. Xie , H. Zhong , et al., “Photo‐Induced Non‐Volatile VO2 Phase Transition for Neuromorphic Ultraviolet Sensors,” Nature Communications 13, no. 1 (2022): 1729. 10.1038/s41467-022-29456-5 PMC8975822 35365642

[49] T. Sánchez , S. Amador‐Alvarado , Y. Kumar , D. Ariza‐Flores , M. Basurto‐Pensado , and V. Agarwal , “Tailoring the UV–visible Reflectivity Range of VO2 Thin Films,” Materials Letters 323 (2022): 132541.

[50] B. Mayer , C. Schmit , and A. Grupp et al., “Tunneling Breakdown of a Strongly Correlated Insulating State in VO2 Induced by Intense Multiterahertz Excitation,” Physical Review B 91, no. 23 (2015): 235113.

[51] Q. Shi , W. Huang , T. Lu , et al., “Nanostructured VO2 Film with High Transparency and Enhanced Switching Ratio in THz Range,” Applied Physics Letters 104 (2014): 7.

[52] S. Barzegar‐Parizi and Z. Vafapour , “Dynamically Switchable Sub‐THz Absorber Using VO2 Metamaterial Suitable in Optoelectronic Applications,” IEEE Transactions on Plasma Science 50, no. 12 (2022): 5038–5045.

[53] E. Mohebbi , E. Pavoni , D. Mencarelli , P. Stipa , E. Laudadio , and L. Pierantoni , “Stability, Phonon Calculations, Electronic Structure, and Optical Properties of a VO2 (M) Nanostructure: A Comprehensive Density Functional Theory Study,” Frontiers in Materials 10 (2023): 1145822.

[54] Z. Liang , X. Liao , J. Yu , et al., “Long‐Term Stability and Efficiency of VO2 Nanostructure‐Based Thermochromic Smart Windows,” ACS Applied Nano Materials 7, no. 18 (2024): 21683–21691.

[55] H. Kim , B. S. Mun , C. Park , and H. Ju , “Effect of over‐Oxidized Surface Layer on Metal Insulator Transition Characteristics of VO2 Films Grown by Thermal Oxidation Method,” Current Applied Physics 17, no. 2 (2017): 197–200.

[56] W. Lu , L.‐M. Wong , S. Wang , and K. Zeng , “Local Phenomena at Grain Boundaries: An Alternative Approach to Grasp the Role of Oxygen Vacancies in Metallization of VO2,” Journal of Materiomics 4, no. 4 (2018): 360–367.

[57] E. D. Case , “Thermal Fatigue and Waste Heat Recovery via Thermoelectrics,” Journal of Electronic Materials 41, no. 6 (2012): 1811–1819.

[58] K. E. Pelcher , M. R. Crawley , and S. Banerjee , “Silica‐Shell Encapsulation and Adhesion of VO2 Nanowires to Glass Substrates: Integrating Solution‐Derived VO2 Nanowires Within Thermally Responsive Coatings,” Materials Research Express 1, no. 3 (2014): 035014.

[59] Y. Chen , X. Zeng , J. Zhu , et al., “High Performance and Enhanced Durability of Thermochromic Films Using VO2@ ZnO Core–shell Nanoparticles,” ACS Applied Materials & Interfaces 9, no. 33 (2017): 27784–27791. 28758388 10.1021/acsami.7b08889

[60] Z. Du , M. Li , F. Zou , et al., “VO_2_@ SiO2 Nanoparticle‐Based Films with Localized Surface Plasmon Resonance for Smart Windows,” ACS Applied Nano Materials 5, no. 9 (2022): 12972–12979.

[61] C. Jiang , L. He , Q. Xuan , Y. Liao , J.‐G. Dai , and D. Lei , “Phase‐Change VO2‐Based Thermochromic Smart Windows,” Light: Science & Applications 13, no. 1 (2024): 255, 10.1038/s41377-024-01560-9. PMC11410829 39294120

[62] T. Chang , X. Cao , N. Li , et al., “ Mitigating Deterioration of Vanadium Dioxide Thermochromic Films by Interfacial Encapsulation.,” Matter 1, no. 3 (2019): 734–744, 10.1016/j.matt.2019.04.004.

[63] M. K. Shahzad , R. Z. A. Manj , and G. Abbas et al., “Influence of VO2 Based Structures and Smart Coatings on Weather Resistance for Boosting the Thermochromic Properties of Smart Window Applications,” RSC Advances 12, no. 48 (2022): 30985–31003. 36349013 10.1039/d2ra04661j PMC9619487

[64] H. T. Dao , S. Sidra , Q. H. Nguyen , M. Mai , P. K. L. Tran , and D. H. Kim , “In Situ Growth and Interfacial Reconstruction of Mo‐Doped Ni3S2/VO2 as Anti‐Corrosion Electrocatalyst for Long‐Term Durable Seawater Splitting,” Applied Catalysis B: Environment and Energy 365 (2025): 124925.

[65] K. Yang , Z. Yin , J. Wu , et al., “Ultra‐Low Power Resistive Random‐Access Memory Based on VO2/TiO2 Nanotubes Composite Film,” Vacuum 216 (2023): 112472.

[66] Z. Cheng , R. Wang , Y. Cao , Z. Cai , Z. Zhang , and Y. Huang , “Intelligent off/on Switchable Microwave Absorption Performance of Reduced Graphene Oxide/VO2 Composite Aerogel,” Advanced Functional Materials 32, no. 40 (2022): 2205160.

[67] P. Prabhathan , K. V. Sreekanth , J. Teng , et al., “Roadmap for Phase Change Materials in Photonics and beyond,“iScience 26, no. 10 (2023), 10.1016/j.isci.2023.107946. PMC10579438 37854690

[68] Y. Zhang , J. B. Chou , J. Li , et al., “Broadband Transparent Optical Phase Change Materials for High‐Performance Nonvolatile Photonics,” Nature Communications 10, no. 1 (2019): 4279, 10.1038/s41467-019-12196-4. PMC6768866 31570710

[69] K. Aryana , H. J. Kim , C. C. Popescu , et al., “Toward Accurate Thermal Modeling of Phase Change Material‐Based Photonic Devices,” Small 19, no. 50 (2023): 2304145. 10.1002/smll.202304145 37649187

[70] L. W. Da Silva , M. Kaviany , and C. Uher , “Thermoelectric Performance of Films in the Bismuth‐Tellurium and Antimony‐Tellurium Systems,” Journal of Applied Physics 97, no. 11 (2005), 10.1063/1.1914948.

[71] J. Pan , H. Hu , Z. Li , J. Mu , Y. Cai , and H. Zhu , “Recent Progress in Two‐Dimensional Materials for Terahertz Protection,” Nanoscale Advances 3, no. 6 (2021): 1515–1531, 10.1039/d0na01046d. 36132557 PMC9419147

[72] A. Kuzikova , L. Shelukhin , F. Maksimov , A. Chernov , R. Pisarev , and A. Kalashnikova , “Laser‐Driven First‐Order Spin Reorientation and Verwey Phase Transitions in Magnetite Fe 3 O. 4 beyond the Range of Thermodynamic Equilibrium,” Physical Review B 107, no. 2 (2023): 024413.

[73] C. Cao , S. Xue , F. Liu , et al., “Studies on the Light‐Induced Phase Transition of CsPbBr3 Metal Halide Perovskite Materials,” ACS Omega 8, no. 22 (2023): 20096–20101. 37305233 10.1021/acsomega.3c02378 PMC10249393

[74] Y. Liu , W. Lv , J. Feng , et al., “Emerging Thermochromic Perovskite Materials: Insights into Fundamentals, Recent Advances and Applications,” Advanced Functional Materials 34, no. 37 (2024): 2402234, 10.1002/adfm.202402234.

[75] H. Fu , “Review of Lead‐Free Halide Perovskites as Light‐Absorbers for Photovoltaic Applications: From Materials to Solar Cells,” Solar Energy Materials and Solar Cells 193 (2019): 107–132, 10.1016/j.solmat.2018.12.038.

[76] I. P. Franco , M. Morales‐Masis , I. Mora‐Seró , and R. Vidal , “Comparative Life Cycle Assessment of Lead‐Free Halide Perovskite Composites/Polymer for Piezoelectric Energy Harvesting,” Sustainable Energy & Fuels 9, no. 16 (2025): 4375–4391. 40655874 10.1039/d5se00717h PMC12242721

[77] C.‐Y. Kim , T. Slusar , J. Cho , and H.‐T. Kim , “Mott Switching and Structural Transition in the Metal Phase of VO2 Nanodomain,” ACS Applied Electronic Materials 3, no. 2 (2021): 605–610, 10.1021/acsaelm.0c00983.

[78] R. Matos and N. Pala , “A Review of Phase‐Change Materials and Their Potential for Reconfigurable Intelligent Surfaces,” Micromachines 14, no. 6 (2023): 1259. 37374844 10.3390/mi14061259 PMC10302041

[79] D. Sahoo and R. Naik , “GSST Phase Change Materials and Its Utilization in Optoelectronic Devices: A Review,” Materials Research Bulletin 148 (2022): 111679.

[80] M. Bohra , N. Agarwal , and V. Singh , “A Short Review on Verwey Transition in Nanostructured Fe3O4 Materials,” Journal of Nanomaterials 2019, no. 1 (2019): 8457383.

[81] X. Zhang , S. Zhang , Z. Ren , et al., “Recent Advances toward Intraoctahedral Phase Change in Metal Halide Perovskite Nanomaterials,” Iscience 27, no. 9 (2024). 10.1016/j.isci.2024.110794 PMC11408066 39297174

[82] T. D. Vu , Z. Chen , X. Zeng , et al., “Physical Vapour Deposition of Vanadium Dioxide for Thermochromic Smart Window Applications,” Journal of Materials Chemistry C 7, no. 8 (2019): 2121–2145.

[83] A. Rakshit , K. Islam , R. Sultana , and S. Chakraborty , “Effect of Oxygen Content and Crystallization Temperature on the Insulator‐to‐Metal Transition Properties of Vanadium Oxide Thin‐Films,” Vacuum 180 (2020): 109633.

[84] R. E. Marvel , R. R. Harl , V. Craciun , B. R. Rogers and R.F. Haglund , “Influence of Deposition Process and Substrate on the Phase Transition of Vanadium Dioxide Thin Films,” Acta Materialia 91 (2015): 217–226.

[85] G. J. Kovács , D. Bürger , I. Skorupa , H. Reuther , R. Heller , and H. Schmidt , “Effect of the Substrate on the Insulator–metal Transition of Vanadium Dioxide Films,” Journal of Applied Physics 109, no. 6 (2011).

[86] Q. Su , C. Huang , Y. Wang , et al., “Formation of Vanadium Oxides with Various Morphologies by Chemical Vapor Deposition,” Journal of Alloys and Compounds 475, no. 1‐2 (2009): 518–523.

[87] P. Kanoo , A. C. Ghosh , and T. K. Maji , “A Vanadium (VO2+) Metal–organic Framework: Selective Vapor Adsorption, Magnetic Properties, and use as a Precursor for a Polyoxovanadate,” Inorganic Chemistry 50, no. 11 (2011): 5145–5152. 21557566 10.1021/ic200463k

[88] H.‐T. Zhang , L. Zhang , D. Mukherjee , et al., “Wafer‐Scale Growth of VO2 Thin Films Using a Combinatorial Approach,” Nature Communications 6, no. 1 (2015): 8475, 10.1038/ncomms9475. PMC4633718 26450653

[89] T. Chirayil , P. Y. Zavalij , and M. S. Whittingham , “Hydrothermal Synthesis of Vanadium Oxides. Chemistry of Materials, 10, no. 10 (1998): 2629–2640.

[90] T.‐D. Nguyen and T.‐O. Do , “Solvo‐Hydrothermal Approach for the Shape‐Selective Synthesis of Vanadium Oxide Nanocrystals and Their Characterization,” Langmuir : the Acs Journal of Surfaces and Colloids 25, no. 9 (2009): 5322–5332. 19301841 10.1021/la804073a

[91] S.‐Y. Liao , X.‐Y. Wang , H.‐P. Huang , et al., “Intelligent Shielding Material Based on VO2 with Tunable Near‐Field and Far‐Field Electromagnetic Response,” Chemical Engineering Journal 464 (2023): 142596.

[92] M. Taha , S. Balendhran , P. C. Sherrell , et al., “Infrared Modulation via Near‐Room‐Temperature Phase Transitions of Vanadium Oxides & Core–Shell Composites,” Journal of Materials Chemistry A 11, no. 14 (2023): 7629–7638.

[93] L. Chen , J. Huang , N. Li , et al., “Broadband Nonlinear Optical Modulator Enabled by VO2/V2O5 Core–shell Heterostructures,” Nanophotonics 11, no. 12 (2022): 2931–2938. 39634087 10.1515/nanoph-2022-0142 PMC11501873

[94] Y. Ko , S. Kang , Y. Yang , J. Lee , and H.‐G. Hur , “Green Synthesis of Vanadium Dioxide Nanoparticles by Shewanella Sp Strain HN‐41,” Journal of Microbiology and Biotechnology 35 (2025): e2502051. 40443232 10.4014/jmb.2502.02051 PMC12149396

[95] S. Genchi , M. Yamamoto , K. Shigematsu , et al., “Growth of Vanadium Dioxide Thin Films on Hexagonal Boron Nitride Flakes as Transferrable Substrates,” Scientific Reports 9, no. 1 (2019): 2857. 30814545 10.1038/s41598-019-39091-8 PMC6393539

[96] M. R. M. Hashemi , S.‐H. Yang , T. Wang , N. Sepúlveda , and M. Jarrahi , “Electronically‐Controlled Beam‐Steering through Vanadium Dioxide Metasurfaces,” Scientific Reports 6, no. 1 (2016): 35439. 27739471 10.1038/srep35439 PMC5064393

[97] Z. Peng , Z. Zheng , Z. Yu , et al., “Broadband Absorption and Polarization Conversion Switchable Terahertz Metamaterial Device Based on Vanadium Dioxide,” Optics & Laser Technology 157 (2023): 108723.

[98] A. Tripathi , J. John , S. Kruk , et al., “Tunable Mie‐Resonant Dielectric Metasurfaces Based on VO2 Phase‐Transition Materials,” ACS Photonics 8, no. 4 (2021): 1206–1213, 10.1021/acsphotonics.1c00124.

[99] D. Wang , L. Yang , B. Cai , et al., “Temperature Tunable Broadband Filter Based on Hybridized Vanadium Dioxide (VO2) Metasurface,” Journal of Physics D: Applied Physics 58, no. 3 (2024): 035106.

[100] D. Yang , W. Wang , E. Lv , et al., “Programmable VO2 Metasurface for Terahertz Wave Beam Steering,” IScience 25 (2022): 8. 10.1016/j.isci.2022.104824 PMC9382261 35992076

[101] Q. Xi , Y. Li , X. Li , Q. Zhang , B. Zhang , and L. Ran , “Wide‐Angle Beam Steering Achieved by an Array‐Fed Phase Reconfigurable Metasurface,” Microwave and Optical Technology Letters 66, no. 1 (2024): e33807, 10.1002/mop.33807.

[102] X. Zhuang , W. Zhang , K. Wang , et al., “Active Terahertz Beam Steering Based on Mechanical Deformation of Liquid Crystal Elastomer Metasurface,” Light: Science & Applications 12, no. 1 (2023): 14, 10.1038/s41377-022-01046-6. PMC9810742 36596761

[103] J. Shabanpour , S. Beyraghi , and A. Cheldavi , “Ultrafast Reprogrammable Multifunctional Vanadium‐Dioxide‐Assisted Metasurface for Dynamic THz Wavefront Engineering,” Scientific Reports 10, no. 1 (2020): 8950. 32488027 10.1038/s41598-020-65533-9 PMC7265406

[104] R. Kargar , K. Rouhi , and A. Abdolali , “Reprogrammable Multifocal THz Metalens Based on Metal–insulator Transition of VO2‐Assisted Digital Metasurface,” Optics Communications 462 (2020): 125331.

[105] S. Y. Liao , X. Y. Wang , Y. Y. Shi , et al., “Reversible Switching between Microwave Absorption and EMI Shielding of VO2 Composite Foam,” Small 20, no. 36 (2024): 2402841. 10.1002/smll.202402841 38693072

[106] S. Prakash , P. Pitchappa , P. Agrawal , et al., “Electromechanically Reconfigurable Terahertz Stereo Metasurfaces, “Advanced Materials 36, no. 32 (2024): 2402069, 10.1002/adma.202402069. 38815130

[107] X. Sun , Z. Qu , J. Yuan , and Q. Wang , “Reconfigurable Broadband Polarisation Conversion Metasurface Based on VO_2_” Photonics and Nanostructures ‐ Fundamentals and Applications 50 (2022): 101012, 10.1016/j.photonics.2022.101012.

[108] Q. Wang , S. Zhang , C. Wang , R. Li , T. Cai , and D. Zhang , “Tunable Infrared Optical Switch Based on Vanadium Dioxide,” Nanomaterials 11, no. 11 (2021): 2988. 34835752 10.3390/nano11112988 PMC8623528

[109] Y. Ke , S. Wang , G. Liu , M. Li , T. J. White , and Y. Long , “Vanadium Dioxide: The Multistimuli Responsive Material and Its Applications,” Small 14, no. 39 (2018): 1802025. 10.1002/smll.201802025 30085392

[110] G. Tanyi , D. Peace , M. Taha , et al., “A Thermally Reconfigurable Photonic Switch Utilizing Drop Cast Vanadium Oxide Nanoparticles on Silicon Waveguides,” Advanced Photonics Research 5, no. 7 (2024): 2300295.

[111] M. Samizadeh Nikoo , R. Soleimanzadeh , A. Krammer , et al., “Electrical Control of Glass‐Like Dynamics in Vanadium Dioxide for Data Storage and Processing,” Nature Electronics 5, no. 9 (2022): 596–603.

[112] Y. Jung , H. Han , A. Sharma , J. Jeong , S. S. Parkin , and J. K. Poon , “Integrated Hybrid VO_2_–silicon Optical Memory,” ACS Photonics 9, no. 1 (2022): 217–223.

[113] I. O. Oguntoye , S. Padmanabha , M. Hinkle , T. Koutsougeras , A. J. Ollanik , and M. D. Escarra , “Continuously Tunable Optical Modulation Using Vanadium Dioxide Huygens Metasurfaces,” ACS Applied Materials & Interfaces 15, no. 34 (2023): 41141–41150. 37606065 10.1021/acsami.3c08493 PMC10472332

[114] Y. Liao , Y. Fan , and D. Lei , “Thermally Tunable Binary‐Phase VO2 Metasurfaces for Switchable Holography and Digital Encryption,” Nanophotonics 13, no. 7 (2024): 1109–1117. 39634017 10.1515/nanoph-2023-0824 PMC11501404

[115] S. D. Ha , Y. Zhou , C. J. Fisher , S. Ramanathan , and J. P. Treadway , “Electrical Switching Dynamics and Broadband Microwave Characteristics of VO2 Radio Frequency Devices,” Journal of Applied Physics 113 (2013): 18.

[116] Z. Gan , F. Chen , Q. Li , et al., “Reconfigurable Optical Physical Unclonable Functions Enabled by VO2 Nanocrystal Films,” ACS Applied Materials & Interfaces 14, no. 4 (2022): 5785–5796. 35044155 10.1021/acsami.1c20803

[117] Y. Zhang , X. Hao , X. Lu , et al., “Tunable C4‐Symmetry‐Broken Metasurfaces Based on Phase Transition of Vanadium Dioxide (VO2),” Materials 17, no. 6 (2024): 1293. 38541446 10.3390/ma17061293 PMC10972120

[118] M. Chen , Z. Xiao , X. Lu , F. Lv , Z. Cui , and Q. Xu , “Dynamically Tunable Multi‐Resonance and Polarization‐Insensitive Electromagnetically Induced Transparency‐Like Based on Vanadium Dioxide Film,” Optical Materials 102 (2020): 109811.

[119] D. Ke , X. Meng , X. H. Rong , C. A. Yu , L. Yu , and D. J. Jia , “Study on Electromagnetically Induced Transparency Effects in Dirac and VO _2 Hybrid Material Structure,” *arXiv preprint* *arXiv:2312.11814* (2023).

[120] X. Duan , S. Chen , H. Cheng , Z. Li , and J. Tian , “Dynamically Tunable Plasmonically Induced Transparency by Planar Hybrid Metamaterial,” Optics Letters 38, no. 4 (2013): 483–485. 23455110 10.1364/OL.38.000483

[121] B. Ko , T. Badloe , and J. Rho , “Vanadium Dioxide for Dynamically Tunable Photonics,” Chemnanomat: Chemistry of Nanomaterials for Energy, Biology and More 7, no. 7 (2021): 713–727.

[122] J. Li , Y. Yang , J. Li , et al., “All‐Optical Switchable Vanadium Dioxide Integrated Coding Metasurfaces for Wavefront and Polarization Manipulation of Terahertz Beams,” Advanced Theory and Simulations 3, no. 1 (2020): 1900183.

[123] Y. Han , L. Duan , and R. Zhang , “Jamming‐Assisted Eavesdropping over Parallel Fading Channels,“ IEEE Transactions on Information Forensics and Security 14, no. 9 (2019): 2486–2499.

[124] S. Zhao , L. Li , C. Hu , et al., “Multiphysical Field Modulated VO2 Device for Information Encryption,” Advanced Science 10, no. 21 (2023): 2300908. 37114834 10.1002/advs.202300908 PMC10375123

[125] L. Li , T. Zhou , Y. Xiao , et al., “Dimension‐Controlled VO2 Film for Optoelectronic Logic Gates and Information Encryption,” ACS Applied Materials & Interfaces 16, no. 31 (2024): 41072–41079. 39046366 10.1021/acsami.4c04546

[126] C. Li , C. Cao , H. Luo , P. Jin , and X. Cao , “Temperature‐Adaptive Radiative Modulator for Multi‐Domain Safety Applications,” Device 2, no. 8 (2024).

